# Differences of resting fMRI and cognitive function between drug-naïve bipolar disorder and schizophrenia

**DOI:** 10.1186/s12888-022-04301-7

**Published:** 2022-10-21

**Authors:** Jiaquan Liang, Wei Huang, Huagui Guo, Weibin Wu, Xiaoling Li, Caixia Xu, Guojun Xie, Wensheng Chen

**Affiliations:** 1 Department of Psychiatry, The Third People’s Hospital of Foshan, Guangdong, People’s Republic of China; 2 Center on Translational Neuroscience, Minzu University of China, Beijing, People’s Republic of China

**Keywords:** Bipolar disorder, Schizophrenia, FALFF, Rs-fMRI, Cognition

## Abstract

**Background:**

Bipolar disorder (BD) and schizophrenia (SC) have many similarities in clinical manifestations. The acute phase of BD has psychotic symptoms, while SC also has emotional symptoms during the onset, which suggests that there is some uncertainty in distinguishing BD and SC through clinical symptoms.

**Aim:**

To explore the characteristics of brain functional activities and cognitive impairment between BD and SC.

**Methods:**

Repeatable Battery for the Assessment of Neuropsychological Status (RBANS) test was performed on patients in drug-naïve BD and SC (50 subjects in each group), and resting-state functional magnetic resonance imaging (rs-fMRI) scanning was performed meanwhile. Rs-fMRI data were routinely preprocessed, and the value of the fractional amplitude of low-frequency fluctuation (fALFF) was calculated. Then each part of the scores of the RBANS and the characteristics of brain function activities were compared between the two groups. Finally used Pearson correlation to analyze the correlation between cognition and brain function.

**Results:**

(1) Compared with BD group, all parts of RBANS scores in SC group decreased; (2) The left inferior occipital gyrus (IOG, peak coordinates − 30, -87, -15; *t* = 4.78, voxel size = 31, Alphasim correction) and the right superior temporal gyrus (STG, peak coordinates 51, -12, 0; *t* = 5.08, voxel size = 17, AlphaSim correction) were the brain areas with significant difference in fALFF values between BD and SC. Compared with SC group, the fALFF values of the left IOG and the right STG in BD group were increased (*p* < 0.05); (3) Pearson correlation analysis showed that the visuospatial construction score was positively correlated with the fALFF values of the left IOG and the right STG (*r*_left IOG_ = 0.304, *p* = 0.003; *r*_right STG_ = 0.340, *p* = 0.001); The delayed memory (figure recall) score was positively correlated with the fALFF value of the left IOG (*r*_left IOG_ = 0.207, *p* = 0.044).

**Discussion:**

The cognitive impairment of SC was more serious than BD. The abnormal activities of the left IOG and the right STG may be the core brain region to distinguish BD and SC, and are closely related to cognitive impairment, which provide neuroimaging basis for clinical differential diagnosis and explore the pathological mechanism of cognitive impairment.

## Introduction

Bipolar disorder (BD) includes type I, defined as manic episode, and type II, defined as one or more hypomanic episodes and severe depressive episode. It is a group of complex and serious chronic mental disorders [1] with cognitive impairment [2, 3]. Schizophrenia (SC) is a mental disease characterized by uncoordinated thinking, cognition, emotion and behavior [4]. It is generally agreed that cognitive impairment is a typical feature of SC [5, 6]. At the same time, the cognitive impairment characteristics of BD are different from those of SC [7–9]. Although the clinical manifestations of the two diseases are different, they also have similarities. Some BD patients have psychotic symptoms in the acute phase [10, 11]. As a subtype of BD, its prognosis is generally worse than that of non-psychotic BD, and its recurrence rate is higher [12]. Meanwhile, SC has emotional symptoms during the onset [13], witch suggests that sometimes it is difficult to distinguish BD and SC from clinical symptoms. At present, the research on the etiology and pathogenesis of BD and SC is still a hot spot in the field of psychiatry. Finding neurobiological markers for the diagnosis and differentiation of the two diseases has always been the direction of efforts [14–16].

In recent years, advances in rs-fMRI have rapidly developed. Voxel-based analyses, especially for fALFF, can well reflect the spontaneous activities of neurons by the changes of blood flow signals accurately in a quiet state noninvasively [17]. Previous neuroimaging studies have shown that patients with BD had structural and functional abnormalities in the prefrontal, temporal, insular and marginal lobe [18–20]. The functional activities of the prefrontal cortex and subcortical related brain networks (cingulate gyrus, insula, striatum, etc.) of SC were abnormal [21, 22]. Correlation analysis also found that both BD and SC had abnormal activities in some specific brain regions and were closely related to cognitive impairment, but the research conclusions were inconsistent [23–26].

Therefore we combined cognitive function evaluation and functional imaging methods, analyzed the characteristics of cognitive impairment and neuroimaging differences between drug-naïve BD and SC patients, in order to provide the theoretical basis for the clinical diagnosis and differentiation of BD and SC.

## Method

### Participants

They were BD/SC patients inpatient or outpatient treated in the department of psychiatry, the Third People’s Hospital of Foshan, Guangdong, People’s Republic of China from July 2016 to September 2021. The acquisition of clinically relevant information was realized through interviews with clinicians. Inclusion criteria: (1) the diagnosis of BD/SC based on the Structured Clinical Interview for DSM-IV-TR (SCID) criteria; (2) 18 ≤ age(years) ≤ 45; (3) education ≥ 9 years; (4) Han nationality, right-handed; (5) drug-naïve, course of disease ≥ 6 months. Exclusion criteria: other mental diseases, brain organic and physical diseases, family history of mental diseases, substance (drugs, alcohol) abuse, brain trauma, neurological diseases, etc.

All subjects volunteered to participate in this study and excluded the contraindications of magnetic resonance imaging (MRI). This study was approved by the ethics committee of the Third People’s Hospital of Foshan, China and the experiments were conducted following the declaration of Helsinki. We obtained written informed consent from all patients before scanning.

### Assessments

Scale evaluation and cognitive test: (1) Positive and Negative Syndrome Scale (PANSS) was used to evaluate the mental symptoms of the SC group. Hamilton Depression Scale-24items (HAMD), Hamilton Anxiety Scale (HAMA) and Bech-Rafaelsen Mania Rating Scale (BRMS) were used to evaluate the BD group. (2) before the assessment of cognitive function, we explained that this cognitive test had no adverse impact on them to eliminate their psychological burdens. The evaluation rules were explained and demonstrated to the subjects with unified guidance, and then the cognition was evaluated with Repeatable Battery for the Assessment of Neuropsychological Status (RBANS) [27].

Rs-fMRI: all subjects were scanned in the radiology department of the Third People’s Hospital of Foshan. The MRI equipment is signa pioneer 3.0T MR of GE company in the United States. Subjects were told to close their eyes, keep a quiet supine position and keep consciousness clear. Firstly, T1 positioning image scanning was carried out, and the scanning parameters were as follows: time repetition (TR) = 2000 ms, echo time (TE) = 30 ms, flip angle (FA) = 90º, field of view (FOV) = 240 mm * 240 mm, matrix = 64 * 64, layer thickness = 4 mm, number of layers = 36, layer spacing = 1 mm, 250 time point data were collected continuously. High resolution T1 scanning parameters: TR = 8.6 ms, TE = 3.3 ms, FA = 12°, FOV = 256 mm * 256 mm, matrix = 256 * 256, layer thickness = 1 mm, layer spacing = 0 mm, slice number = 172. Data preprocessing: the DPARSF system (based on SPM 8 and REST 1.8 software; http://www.restfmri.net) was used to preprocess all image data. The first 10 sequences of images were deleted to exclude the influence of machine start-up on signal collection, and the remaining 240 sequences were used for data analysis. The preprocessing process included head movement correction and spatial standardization. According to the head movement correction curve, the data of 5 subjects with head movement translation > 2 mm and rotation > 2^°^ were eliminated. The resting functional image was re-registered with T1 image, and then the imaging data were normalized to the Montreal Neurological Institute (MNI) space, and resampled to 3 mm * 3 mm * 3 mm resolution.

FALFF: removed the linear drift of the preprocessed data, divided the energy of each frequency in the low-frequency range (0.01 Hz < f < 0.1 Hz) by the energy of each frequency in the whole frequency range to obtain the fALFF value of each voxel, and divided it by the mean of the whole brain signal amplitude, so as to reduce the difference in the overall level of the whole brain fALFF value.

Data analysis: SPSS 21 (https://www.ibm.com/analytics/spss-statistics- software) was used to analyze the score of clinical scale. SPM 8 software (https://www.fil.ion.ucl.ac.uk/spm/software/spm8) was used to conduct two sample t-test on two groups of standardized fALFF images. The mean value of head movement parameters, gender, age and years of education of each subject were taken as covariates, and the threshold level was set as *p* < 0.001 (Alphasim correction). Finally, Pearson correlation analysis was carried out to test the cognitive function score and the fALFF value of different brain regions.

## Result

### Clinical data and cognitive function test

There was no significant difference between BD and SC in age, gender, years of education and course of disease (*p* < 0.05). While there were significant differences in immediate memory (learning and story memory), visuospatial construction, language, attention (digit span), and delayed memory (list Recall, list Recognition, story recall and figure recall), which belong to the RBANS test (*p* < 0.05). Compared with BD, the cognition of SC generally decreased (*p* < 0.05). (Table 1)

**Table 1:** Clinical data and RBANS test between BD and SC

	BD	SC	*Χ*^*2*^ /*t*	*p*
Participants	50	50	-	-
Age (years)	30.80 ± 9.56	33.28 ± 6.44	1.52	0.131
Gender (m/f)	23 / 27	17 / 33	1.50	0.307
Education	12.28 ± 2.86	11.42 ± 2.921	1.49	0.139
Course of disease (w)	37.18 ± 15.93	39.80 ± 15.56	0.591	0.557
Psychotic symptoms	21	50	-	-
Manic/depressive episode (n)	13/37	-	-	-
HAMD	16.66 ± 8.11	-	-	-
HAMA	11.86 ± 6.77	-	-	-
BRMS	15.12 ± 8.03	-	-	-
PANSS	-	65.36 ± 19.44	-	-
RBANS				
Immediate memory (Learning)	21.66 ± 5.52	16.50 ± 6.84	4.01	<0.001
Immediate memory (Story Memory)	9.04 ± 4.35	5.02 ± 3.80	4.74	<0.001
Visuospatial Construction	16.12 ± 4.66	15.78 ± 3.96	2.72	0.008
Language	16.11 ± 4.05	11.50 ± 4.41	5.25	<0.001
Attention (Digit span)	12.83 ± 2.51	10.50 ± 2.51	4.47	<0.001
Attention (Coding)	40.32 ± 13.33	27.04 ± 13.32	4.81	<0.001
Delayed memory (List Recall)	4.62 ± 2.35	2.59 ± 2.54	4.01	<0.001
Delayed memory (List Recognition)	18.51 ± 1.88	17.07 ± 3.06	2.75	0.007
Delayed memory (Story Recall)	4.23 ± 2.90	2.35 ± 2.36	3.44	0.001
Delayed memory (Figure Recall)	10.84 ± 4.10	8.78 ± 5.62	2.05	0.044

Note: BD bipolar disorder; SC schizophrenia; HAMD Hamilton Depression Scale-24items; HAMA Hamilton Anxiety Scale; BRMS Bech-Rafaelsen Mania Rating Scale; PANSS Positive and Negative Syndrome Scale; RBANS Repeatable Battery for the Assessment of Neuropsychological Status; Values are expressed as mean ± standard deviation

### FALFF

**Fig. 1:**
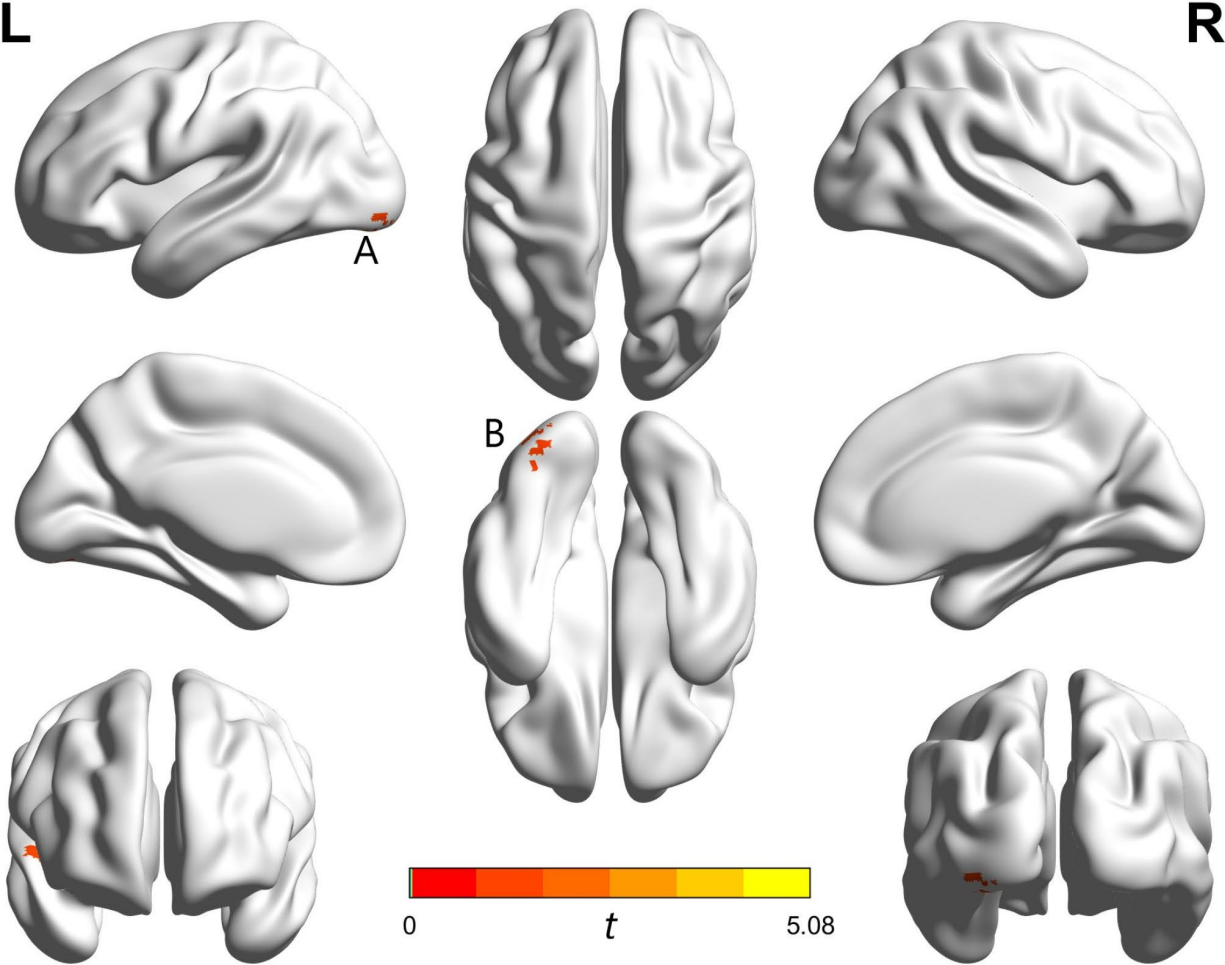
FALFF abnormalities in the BD compared with SC. (A) The left inferior occipital gyrus (IOG), *t* = 4.78, cluster size = 31; (B) The right superior temporal gyrus (STG), *t* = 5.08, cluster size = 17; *p* < 0.05, AlphaSim correction

Compared with SC, the brain areas with significant differences in BD were the left inferior occipital gyrus (IOG, peak coordinates of − 30, − 87, − 15; *t* = 4.78, voxel size = 31, *p* < 0.05, Alphasim correction) and the right superior temporal gyrus (STG, peak coordinates of 51, − 12, 0; *t* = 5.08, voxel size = 17, *p* < 0.05, Alphasim correction). (Fig. 1)

### Pearson correlation analysis

**Fig. 2:**
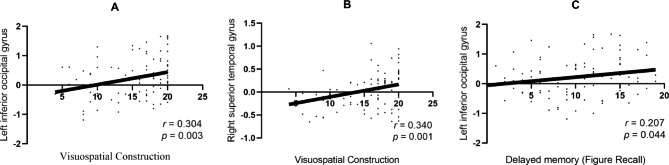
**A/B** The fALFF values of the left IOG and the right STG were positively correlated with visuospatial construction score (*r*_A_ = 0.30, ,*p*_A_ = 0.003; *r*_B_ = 0.34, *p*_B_ = 0.001); **C** The fALFF values of the left IOG were positively correlated with delayed memory (Figure Recall) score (*r*_C_ = 0.20, *p*_C_ = 0.044)

The fALFF values of the left IOG and the right STG were positively correlated with visuospatial construction score (*r*_left IOG_ = 0.304, *p* = 0.003; *r*_right STG_ = 0.340, *p* = 0.001). The fALFF values of the left IOG were positively correlated with delayed memory (Figure Recall) score (*r*_left IOG_ = 0.207, *p* = 0.044). (Fig. 2)

## Discussion

At present, it is generally believed that BD and SC have cognitive impairment in the course of disease to some extent [28–30]. The difference in cognition is of great significance in the differential diagnosis between them. Previous studies had found that SC performed worse than BD in executive function, working memory, IQ, association, attention concentration and perceptual motor function [31–33]. Our study showed that the cognitive test score of SC was lower than BD, which was partially similar to previous studies, suggested that the cognitive impairment of SC was more serious than BD. And although BD and SC sometimes have the same cognitive impairment, their recovery after treatment is still different [34]. A longitudinal study suggested that after standardized clinical treatment, BD patients could get cognitive recovery, left slight cognitive damage, and could be repaired to a certain extent through follow-up cognitive exercise [35]. However, the cognitive impairment of SC was usually considered irreversible because it was considered to be related to nerve injury [36–39]. Therefore, cognitive testing is helpful to distinguish the diagnosis of these two diseases.

Our study also showed that the fALFF values of the left IOG and the right STG in BD were higher than SC. According to Zhang et al’s study [40], there were a large number of nerve fiber connections between temporal and occipital lobes, which would be damaged by neurological and mental diseases. Meanwhile, the results of an Asian population study suggested that SC has significant functional impairment in the temporal and occipital lobes[41]. The dysfunction of these brain regions was related to the dysfunction of language, attention and higher-level visual and auditory comprehensive processing [42]. What’s more, emotional perception was an effective tool to distinguish SC from BD, and the frontotemporal occipital circuit was considered to be related to impaired emotional perception [25]. Ehrminger et al’s research indicated that the impairment of cognitive function was partly reversible in the course of BD [35]. Therefore, it can be understood that the signal activity of the BD brain area is more active than that of SC’s, which also supports ours results.

We made a correlation analysis between the cognitive scores in RBANS and the fALFF values of the left IOG and the right STG. The results showed that there was a significant positive correlation between the visual construction score and the abnormal activities of that two brain regions. Previous studies had suggested that SC has significant impairment of visual breadth cognitive function [43–45], which would affect the prognosis of patients [46], and which might also be one of the factors for the poor prognosis of SC compared with BD. Therefore, the evaluation of visual construction cognition plays an important role in the diagnosis and prognosis of SC. Besides, We also found that the delayed memory (figure recall) score was closely related to the brain function signals of the left SOG. As we know, the occipital lobe has extensive connections with other regions of the two cerebral hemispheres and plays an important role in the integration of visual information with information gathered by other sensory systems. At the same time, it also connects visual information with brain processing systems of other executive functions. The study indicated that the microstructure of frontal occipital white matter bundle was closely related to object working memory. When its microstructure was damaged (such as multiple sclerosis and other diseases), object working memory would be affected [47], which confirmed our results from the side.

In conclusion, this study suggests that cognitive function test can be used as one of the important differential indexes between BD and SC in clinic. The abnormal activities of the left IOG and the right STG may be the core brain region to distinguish BD and SC, and are closely related to cognitive impairment, which provide neuroimaging basis for clinical differential diagnosis and explore the pathological mechanism of cognitive impairment.

At the same time, there are some limitations in this study: BD and SC have many similarities in the early stage of onset. In order to reduce the possibility of diagnostic errors, we had to limit the course of the patient to more than 6 months and exclude the patients who are seriously ill and can not cooperate to complete the test, which might skew the results of the data.

In addition, this study only analyzed the characteristics of low-frequency amplitude activity of brain function, which can be further discussed in combination with other brain function indexes, such as regional homogeneity, degree centrality, functional connection and so on.

## Data Availability

The datasets generated and/or analyzed during the current study are not publicly available due to confidentiality but are available from the corresponding author upon reasonable request.
